# High doses of rosuvastatin induce impaired branched‐chain amino acid catabolism and lead to insulin resistance

**DOI:** 10.1113/EP090305

**Published:** 2023-05-04

**Authors:** Xue Bai, Xingzhen Long, Fang Song, Baolin Chen, Changcheng Sheng, Cailin Tang, Li Li, Jiaxing Zhang, Rui Zhang, Jiquan Zhang, Jiali Li

**Affiliations:** ^1^ Department of Pharmacy Guizhou Provincial People's Hospital Guiyang Guizhou China; ^2^ The First Affiliated Hospital Guizhou University of Traditional Chinese Medicine Guiyang Guizhou China; ^3^ Department of Cardiology Guizhou Provincial People's Hospital Guiyang Guizhou China; ^4^ State Key Laboratory of Functions and Applications of Medicinal Plants & College of Pharmacy Guizhou Provincial Engineering Technology Research Center for Chemical Drug R&D Guizhou Medical University Guiyang Guizhou China; ^5^ Institute of Clinical Pharmacology, School of Pharmaceutical Sciences Sun Yat‐Sen University Guangzhou Guangdong China

**Keywords:** Akt, BCAA catabolism, GSK3β, insulin resistance, PP2Cm, rosuvastatin

## Abstract

Accumulating evidence indicates that patients treated with rosuvastatin have an increased risk of developing new‐onset diabetes. However, the underlying mechanism remains unclear. In this study, we administered rosuvastatin (10 mg/kg body weight) to male C57BL/6J mice for 12 weeks and found that oral rosuvastatin dramatically reduced intraperitoneal glucose tolerance. Rosuvastatin‐treated mice showed considerably higher serum levels of branched‐chain amino acids (BCAAs) than control mice. They also showed dramatically altered expression of BCAA catabolism‐related enzymes in white adipose tissue and skeletal muscle, including downregulated mRNA expression of *BCAT2* and protein phosphatase 2Cm (*PP2Cm*) and upregulated mRNA expression of branched‐chain ketoacid dehydrogenase kinase (*BCKDK*). The levels of BCKD in the skeletal muscle were reduced in rosuvastatin‐treated mice, which was associated with lower PP2Cm protein levels and increased BCKDK levels. We also investigated the effects of rosuvastatin and insulin administration on glucose metabolism and BCAA catabolism in C2C12 myoblasts. We observed that incubation with insulin enhanced glucose uptake and facilitated BCAA catabolism in C2C12 cells, which was accompanied by elevated Akt and glycogen synthase kinase 3 β (GSK3β) phosphorylation. These effects of insulin were prevented by co‐incubation of the cells with 25 μM rosuvastatin. Moreover, the effects of insulin and rosuvastatin administration on glucose uptake and Akt and GSK3β signaling in C2C12 cells were abolished when PP2Cm was knocked down. Although the relevance of these data, obtained with high doses of rosuvastatin in mice, to therapeutic doses in humans remains to be elucidated, this study highlights a potential mechanism for the diabetogenic effects of rosuvastatin, and suggests that BCAA catabolism could be a pharmacological target for preventing the adverse effects of rosuvastatin.

## Abstract (highlights)

- **What is the central question of this study?**
  Is there a risk of developing diabetes associated with statin treatment? What is the underlying mechanism of the increased incidence rate of new‐onset diabetes in patients treated with rosuvastatin?
- **What is the main finding and its importance?**
  Rosuvastatin therapy reduced intraperitoneal glucose tolerance and changed the catabolism of branched‐chain amino acid (BCAAs) in white adipose tissue and skeletal muscle. Protein phosphatase 2Cm knockdown completely abolished the effects of insulin and rosuvastatin on glucose absorption. This study provides mechanistic support for recent clinical data on rosuvastatin‐related new‐onset diabetes and underscores the logic for intervening in BCAA catabolism to prevent the harmful effects of rosuvastatin.

## 1 INTRODUCTION

Rosuvastatin, one of the most commonly prescribed drug in the USA, possesses hydrophilic qualities and is more effective than other statins such as pravastatin and simvastatin in lowering cholesterol levels (Paoletti et al., 2001). Due to the elevated cardiovascular risk in selected identified patients, the American College of Cardiology and the American Heart Association issued revised guidelines on the treatment of blood cholesterol, suggesting that statin combination therapy could improve cardiovascular outcomes (Mirbolouk & Blaha, 2020; Toth et al., 2016). Although statins are effective in cardiovascular disease treatment, current research suggests that they elevate the risk of developing new‐onset diabetes (Salunkhe et al., 2016). In a double‐blind randomized study comparing participants allocated to 20 mg rosuvastatin or placebo treatment, the JUPITER trial found an increased risk of developing diabetes (Cederberg et al., 2015). Another research established a link between statin use and an increased risk of developing diabetes, as well as reduced insulin sensitivity and secretion (Mora et al., 2010). However, the mechanisms underlying increased diabetes incidence due to rosuvastatin treatment remain unknown.

Increased levels of circulating branched‐chain amino acids (BCAAs) have been related to insulin resistance in patients with obesity and diabetes (Preiss & Sattar, 2012). The proteinaceous BCAAs are the essential amino acids leucine, isoleucine and valine, which are obtained from dietary proteins (Ridker et al., 2008). Although there exists a beneficial relationship between BCAA‐enriched diets and metabolic health (Yoon, 2016), elevated levels of BCAAs were found to correspond with an increased risk of developing insulin resistance and type 2 diabetes mellitus in both rat models and humans (Zhao et al., 2016). BCAA deficiency impairs normal cell growth and development, whereas blockade of BCAA metabolism catalysed by branched‐chain α‐ketoacid dehydrogenase (BCKD) results in the accumulation of BCAAs and their potentially harmful α‐ketoacid derivatives (branched‐chain α‐ketoacids, BCKA) (Lu et al., 2013). For instance, maple syrup urine disease is an autosomal recessive hereditary metabolic illness characterized by a BCKD complex deficit; it is distinguished by high levels of plasma BCAAs and urine branched‐chain α‐ketoacids (Lynch & Adams, 2014). Therefore, a better understanding of BCAA metabolic pathways could help humans control their glucose metabolism and homeostasis.

The initial step in the catabolism of BCCAs is catalysed by a cytosolic or mitochondrial isoform of branched‐chain aminotransferases, such as branched‐chain amino acid transaminase 2 (BCAT2) (Giesbertz & Daniel, 2016). BCAT1, another BCAT, is a cytoplasmic protein commonly expressed in the brain. Most investigations into BCAT1 have been focused on tumors (Neinast et al., 2019). The BCKD complex, a multimeric mitochondrial enzyme complex consisting of three catalytic components (E1, E2, and E3), catalyses the next and rate‐limiting stage of the BCAA metabolic pathway (Schiff et al., 2016). The BCKD complex in mammals contains 24 copies of dihydrolipoyl transacylase (E2 component, encoded by *DBT*); multiple copies of branched‐chain α‐ketoacid decarboxylase (E1 component), each containing two E1α and E1β subunits (encoded by *BCKDHA* and *BCKDHB*, respectively); and a dihydrolipoamide dehydrogenase (E3 component, encoded by *DLD*) (Ambrus & Adam‐Vizi, 2018). The activity of the BCKD complex is tightly regulated under various growth and nutrient environments to ensure a constant plasma level of BCAAs (Dhanani et al., 2019). The reversible phosphorylation of the E1α subunit (BCKDHA) at the Ser293 residue is a primary mechanism in the control of BCKD activity (Lu et al., 2009). Under low levels of BCAAs, BCKD is inactivated through phosphorylation at the Ser293 residue of BCKDHA by kinases such as BCKDK (Lynch & Adams, 2014). When the levels of BCAAs are high, BCKD is activated by phosphates, dephosphorylating the Ser293 residue of BCKDHA. Protein phosphatase 2Cm (PP2Cm) has been identified as the endogenous BCKD phosphatase required for the nutrient‐mediated regulation of BCKD activity (Lu et al., 2009). Recent studies have reported that dietary supplementation with essential amino acids and a unique branched‐chain amino acid‐enriched mixture (BCAAem) enhanced the positive effects of rosuvastatin on mouse kidney and avoided rosuvastatin myopathy in mice, respectively (Corsetti et al., 2014; D'Antona et al., 2016b). Nevertheless, it is unclear whether and how rosuvastatin affects the catabolism of BCAAs.

In this study, using an in vivo mouse model and an in vitro C2C12 myoblast cell model, we investigated the effects of high doses of rosuvastatin (10 mg/kg body weight in vivo, and 25 μM in vitro) on glucose metabolism and BCAA catabolism. Our findings demonstrated that rosuvastatin therapies cause systemic insulin resistance in mice, which is related to poor BCAA catabolism in vivo and in vitro.

## 2 METHODS

### 2.1 Ethical approval

This study was approved by the Institutional Animal Care and Use Committee of Guizhou Provincial People's Hospital (Approval number: 2020‐20). All animal experiments followed the *Guide for the Care and Use of Laboratory Animals* (2011 Eighth Edition, National Research Council) for the welfare of the laboratory animals. All necessary procedures were implemented to minimize the pain and suffering of animals.

### 2.2 Mice and treatments

Male C57BL/6J mice weighing 18–22 g (aged 4−6 weeks) were purchased from Charles River Laboratories (Beijing, China) and maintained at room temperature (22 ± 1°C) with a 12/12‐h light–dark cycle and unlimited access to food and water ad libitum in a specific pathogen‐free facility at the Animal Center of Guizhou Provincial People's Hospital. Mice were orally administered rosuvastatin (10 mg/kg body weight in 1 ml of water; cat. no. SML1264, Sigma‐Aldrich, St Louis, MO, USA) once a day. Rosuvastatin was first dissolved to a concentration of 50 mM in dimethyl sulfoxide (DMSO, Sigma‐Aldrich) and then diluted in drinking water to the working concentration. The control group was provided the same amount of drinking water without any medication. Mice were killed by CO_2_ inhalation at a rate of 3 l/min in a chamber (20 cm wide × 30 cm deep × 15 cm high) at 12 weeks after rosuvastatin treatments, and this was confirmed by continued CO_2_ exposure for more than 15 min after breath arrest. White adipose tissues, liver and hindlimb soleus skeletal muscle tissues were quickly collected and processed for further analysis after the mice were killed.

### 2.3 Routine blood analyses

Mice were fasted overnight, and a total volume of ∼200 μl blood was collected from the tail vein within one day (each time 10–25 μl). The blood samples were centrifuged at 1000 *g* for 10 min at room temperature to separate serum. The serum samples were diluted 10 times in sterile phosphate‐buffered saline (PBS) if needed, and 20–50 μl of the diluted serum was used for subsequent assays following the protocols of the kit manuals. Fasting blood glucose levels were measured using a kit (Nanjing Jiancheng Bioengineering Institute, cat. no. F006‐1‐1, Nanjing, China). An enzyme‐linked immunosorbent assay (ELISA) kit (cat. no. H203‐1‐2, Nanjing Jiancheng) was used to measure fasting insulin levels in the serum samples. Assay kits provided by Nanjing Jiancheng Bioengineering Institute were used to measure the levels of serum total cholesterol (cat. no. A111‐1‐1), total triglyceride (TG; cat. no. A110‐1‐1), high‐density lipoprotein cholesterol (HDL‐c; cat. no. A112‐1‐1) and free fatty acids (cat. no. A042‐2‐1). The levels of β‐hydroxybutyrate (BHB) in the serum were measured using a kit from Abcam (cat. no. ab83390, Cambridge, UK). All routine blood analyses were conducted according to the manufacturers’ instructions.

### 2.4 BCAA assay

The BCCA content in the serum and culture supernatant samples was measured using a BCAA assay kit (cat. no. ab83374, Abcam) according to the manufacturer's protocol. The kit utilizes an enzyme assay in which BCAAs are oxidatively deaminated, resulting in NADH, which is used to reduce the probe and produces a colored product. Briefly, an equal volume of serum sample was combined with the assay buffer and centrifuged for 10 min at 15,000 *g*. The supernatant sample was combined separately with the enzyme reaction mixture, along with standards with known BCAA concentrations, and the mixtures were incubated at room temperature for 30 min in the dark. Absorbance was measured at 450 nm using a microplate reader (SpectraMax iD3 Hybrid Multi‐Mode Microplate Reader, Molecular Devices, San Jose, CA, USA).

### 2.5 Intraperitoneal glucose tolerance test

All mice were starved overnight the day before the test, and an intraperitoneal glucose tolerance test (IPGTT) was performed 12 weeks after rosuvastatin administration. Mice were administered oral glucose at 2 g/kg body weight in the early morning of the day of the IPGTT measurement after an overnight fast. Blood samples were collected before (baseline, 0 min) and at 15, 30, 60, 90 and 120 min after oral glucose administration. Blood glucose levels were measured as mentioned earlier. The level of plasma insulin was also determined by ELISA (Nanjing Jiancheng).

### 2.6 Cell culture and treatments

C2C12 is a mouse myoblast cell line derived from the American Type Culture Collection (ATCC; Manassas, VA, USA). The provider analysed the cells for mycoplasma contamination and authenticated them using short tandem repeat (STR) DNA profiling. Cryopreserved C2C12 cells were recovered from liquid nitrogen using an AccuVital cell recovery kit (AccuRef Scientific, Xi'an, China) and cultured in Dulbecco's modified Eagle medium (DMEM; cat. no. 11965092, Thermo Fisher Scientific, Waltham, MA, USA) supplemented with 10% fetal bovine serum (FBS; cat. no. 16140071, Thermo Fisher Scientific), 1% l‐glutamine, 100 units/ml of penicillin, and 100 μg/ml of streptomycin (cat. no. 10378016, Thermo Fisher Scientific) in a humidified atmosphere with 5% CO_2_ at 37°C.

The cytotoxicity of rosuvastatin at various concentrations (1, 5, 10, 25, 50 and 100 μM) in C2C12 myoblasts was evaluated using the Cell Counting Kit‐8 assay (AccuRef Scientific), which revealed 25 μM as the acceptable maximum concentration. Rosuvastatin was first dissolved in 100% DMSO (Sigma‐Aldrich) to form a 50 mM stock solution, which was then diluted to working quantities in complete culture mediums. Cells were treated with 100 nM insulin (cat. no. I0320000, Sigma‐Aldrich) for 10 min, with 25 μM rosuvastatin alone for 48 h, or with rosuvastatin for 48 h followed by insulin for 10 min. Then, the cells were subjected to a series of tests. Cells treated with an equivalent volume of 100% DMSO served as controls.

### 2.7 Lentivirus expressing short hairpin RNA

Three short hairpin RNA (shRNA) plasmids and a random negative control (shNC) plasmid were provided by GeneCheme (Shanghai, China). One million HEK293T cells were plated into 100‐mm dishes and cultured at 37°C for 24 h. Next, 10 μg shNC or shPP2Cm‐knockdown lentiviral plasmid (PP2Cm shRNA‐1/2/3) and 7.5 μg helper plasmids were mixed with 0.95 ml of Opti‐MEM (Thermo Fisher Scientific) and 0.05 ml of lipofectamine 3000 (cat. no. L3000015, Thermo Fisher Scientific). After gentle mixing, the solution was diluted with serum‐free DMEM and cultured for 8 h before being replaced with 10 ml of complete DMEM (supplemented with 10% FBS). The lentiviral particles were collected at 24‐h intervals for 96 h. C2C12 myoblasts in the logarithmic growth phase were seeded in six‐well plates with 70% confluency overnight, then the lentiviral supernatants were added to the C2C12 myoblasts at a multiplicity of infection of 30. Cells were used for additional experiments 48 h after virus infection.

### 2.8 BCKDK assay

Hindlimb soleus skeletal muscles were harvested immediately after killing and stored at −80°C until use. The BCKDK level was determined using an ELISA kit (cat. no. SEJ560Ra, Wuhan USCN, Wuhan, China) according to the manufacturer's instructions. Briefly, 100 μl of diluted homogenized skeletal muscle samples, standards, or a blank control were added to each well, and the plate was incubated at 37°C for 2 h. Then, without washing, 100 μl of Detection Reagent A was added to each well, followed by incubation for 1 h at 37°C. After three washes with Wash Solution, 100 μl of Detection Reagent B was added to each well, and the plate was incubated for 30 min at 37°C. After five washes, 90 μl of Substrate Solution was added to each well, followed by incubation for 20 min at 37°C. Absorbance was quickly measured at 450 nm after adding stop solution using a Biotek Synergy H1 microplate reader (BioTek Instruments, Winooski, VT, USA).

### 2.9 Glucose uptake assay

A cell‐based glucose uptake experiment was performed according to the manufacturer's instructions using an Abcam kit (cat. no. ab204702). In a 12‐well plate, 5 × 10^4^ cells were seeded into each well. After 24 h, the fluorescent glucose analogue was added to each sample and incubated for 30 min at 37°C with 5% CO_2_. Thereafter, the cells were trypsinized and collected. The fluorescence intensity of glucose uptake was measured using a flow cytometer (BD FACSCalibur, BD Biosciences, San Jose, USA), and 5 × 10^4^ events were recorded for each sample. The mean fluorescence intensity in the control group was arbitrarily fixed at 100% to demonstrate glucose absorption.

### 2.10 RNA isolation, reverse transcription and quantitative PCR

RNA was isolated using an AccuRef RNA isolation kit (AccuRef Scientific) according to the manufacturer's instruction. Total RNA (1 μg of each sample) was used to synthesize cDNA using the FastKing RT kit (cat. no. KR116, Tiangen Biotech, Beijing, China). Quantitative real‐time PCR (RT‐qPCR) was performed using a standard protocol from the SYBR Green PCR kit (cat. no. FP209, Tiangen Biotech) on an AB7500 RT‐PCR instrument (Thermo Fisher Scientific, USA) under the conditions of 95°C for 4 min, 40 cycles of 95°C for 1 min, 57°C for 30 s, 72°C for 1 min, and 72°C for 10 min. Normalization to *GAPDH* was used for relative determination, and gene expression levels were calculated using the $2^{-\Delta \Delta C_{T}}$ method. The primers used for PCR are listed in Table 1.

**TABLE 1:** The sequences of PCR primers in this study.

Name	Sequences (5′–3′)
*GAPDH*	F: 5′‐TCGCTCCTGGAAGATGGTGAT‐3′;
	R: 5′‐CAGTGGCAAAGTGGAGATTGTTG‐3′
*BCAT2*	F: 5′‐TGAAGGCAAGCAACTCCACA‐3′;
	R: 5′‐GCACTGGCTCCGTACTGAAT‐3′
*BCKDHA*	F: 5′‐TCCAGGGTGACTTTTGCTCC‐3′;
	R: 5′‐TTCCCACCCCTAAAAGGCAC‐3′
*BCKDHB*	F: 5′‐TTGGCTGATGAGCTGCGAAT‐3′;
	R: 5′‐TCTTAAGATCACCGCCCACG‐3′
*DBT*	F: 5′‐GCAGGCCTTGTTAGGTCCAT‐3′;
	R: 5′‐TGTGTTTCCTCCACGCTACC‐3′
*PP2Cm*	F: 5′‐TCTCATGGTTGGAATGGCCC‐3′;
	R: 5′‐CAGGTCACTCGCTCTCAGTG‐3′
*BCKDK*	F: 5′‐CGCTATCAGAGGCCCCAAAA‐3′;
	R: 5′‐ACTGCCCAGTCTAGTGACCT‐3′

Abbreviations: F, forward; R, reverse.

### 2.11 Western blot

Cells were lysed in a protein lysis buffer (Exinno, Xi'an, China) containing a protease and phosphatase inhibitor cocktail (cat. no. PPC1010‐5ML, Sigma‐Aldrich) on ice for 30 min after washing with cold PBS. Proteins in the supernatant samples were quantified using the BCA method (cat. no. A53225, Thermo Fisher Scientific) according to the manufacturer's instructions after centrifugation at 13,000 *g* for 20 min at 4°C. The total proteins were then boiled with a loading buffer, separated using 10% SDS‐PAGE, and transferred to polyvinylidene difluoride membranes (cat. no. 05317‐10EA, Millipore, Billerica, MA, USA) in equal proportions. The membranes were incubated overnight with the indicated primary antibodies after blocking with 5% defatted milk in TBST (100 mM Tris, 166 mM NaCl, 0.5% Tween 20) for 2 h. The secondary antibodies were horseradish peroxidase‐conjugated anti‐rabbit IgG (1:10,000; cat. no. HS101, TransGen Biotech, Beijing, China) or anti‐mouse IgG (1:10,000; cat. no. HS201, TransGen Biotech). Proteins of interest were visualized using an enhanced chemiluminescence kit (AccuRef Scientific). Densitometry was used to determine the band using the ImageJ software (National Institutes of Health, Bethesda, USA). Table 2 lists the information of antibodies used for western blot assays.

**TABLE 2:** Information for primary antibodies used for western blot assays in this study.

Target	Company	cat. no.	Source species	Dilution
*GAPDH*	Abcam	ab9485	Rabbit	1: 2500
*BCKDHA*	Abcam	ab126173	Rabbit	1: 2000
*p‐BCKDHA*	Abcam	ab275961	Rabbit	1: 1000
*PP2Cm*	Abcam	ab224424	Rabbit	1: 100
*BCKDK*	Abcam	ab128935	Rabbit	1: 5000
*Akt*	Abcam	ab8805	Rabbit	1: 500
*p‐Akt*	Abcam	ab38449	Rabbit	1: 750
*GSK3β*	Abcam	ab93926	Mouse	1: 1000
*p‐GSK3β*	Abcam	ab107166	Rabbit	1 μg/ml
*pBCKD E1α*	Cell signaling	40368	Rabbit	1: 1000
*BCKD E1α*	Cell signaling	90198	Rabbit	1: 1000

### 2.12 Statistical analysis

The statistical analysis was conducted using GraphPad Prism 8.0 (GraphPad Software, San Diego, CA, USA). Data from at least three biological repetitions were used to calculate the mean ± standard deviation (SD) of each value. An unpaired Student's *t*‐test was used to analyse differences between the two groups, and comparisons among multiple groups were performed using one‐way, two‐way, and three‐way analysis of variance (ANOVA) with Tukey's *post hoc* test. A *P*‐value of <0.05 was considered to be statistically significant.

## 3 RESULTS

### 3.1 Rosuvastatin induces systemic insulin resistance and increases serum BCAA levels in mice

**FIGURE 1:**
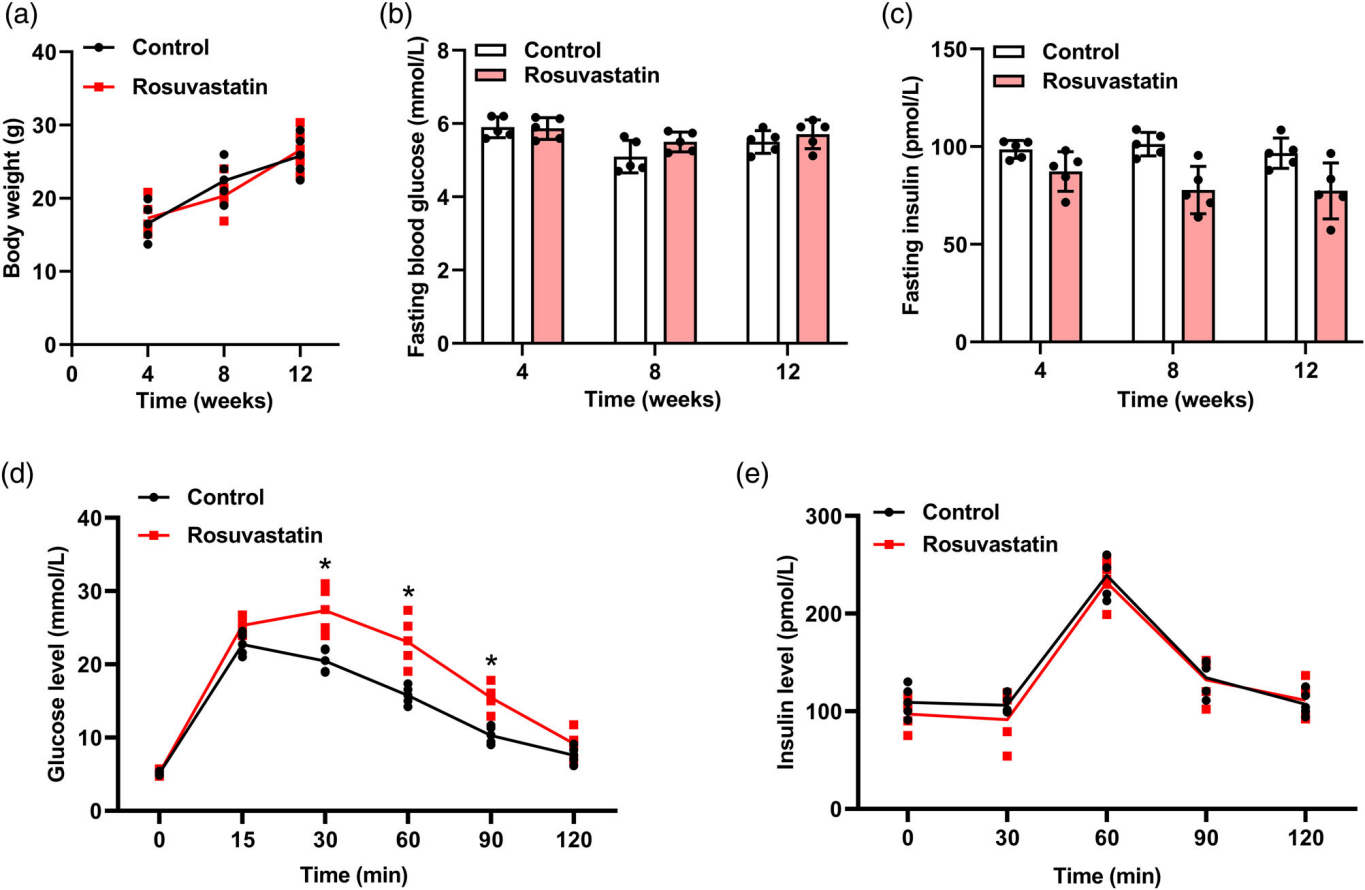
Rosuvastatin induced systemic insulin resistance in mice. (a–d) Mice received daily oral dosing of rosuvastatin (10 mg/kg body weight; rosuvastatin group) or equal amounts of drinking water (control group) for 12 weeks. Blood samples were collected at 4, 8 and 12 weeks after oral administration. (a) Body weights of mice in the two groups displayed at the indicated time points after oral administration. (b, c) Levels of fasting blood glucose (b) and fasting insulin (c) in serum samples obtained from mice at the indicated time points. (d) At 12 weeks after oral dosing, mice in the two groups were subjected to an intraperitoneal glucose tolerance test (IPGTT). The blood glucose levels before (0 min) and at 15, 30, 60, 90 and 120 min after oral glucose administration were measured. (e) Plasma insulin at 12 weeks after oral dosing in the two groups. *n* = 5 mice for each group; *P < 0.05, between the indicated groups.

**FIGURE 2:**
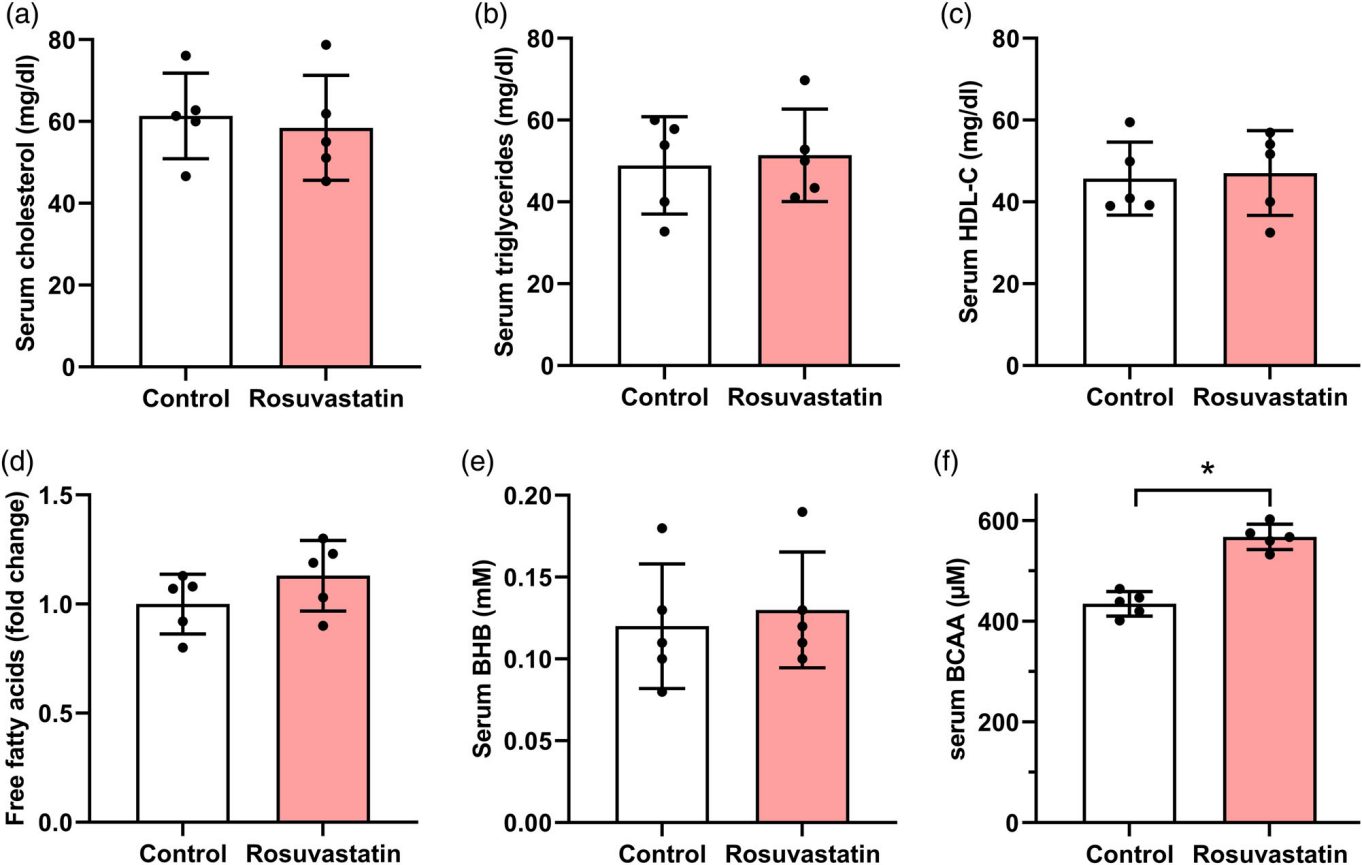
General metabolic profiling in blood samples collected from mice administered oral rosuvastatin. (a–f) Levels of serum cholesterol (a), triglyceride (b), high‐density lipoprotein cholesterol (HDL‐c; c), free fatty acids (d), β‐hydroxybutyrate (BHB; e) and branched‐chain amino acids (BCAAs; f) in mice at 12 weeks after oral dosing of rosuvastatin (rosuvastatin group) or drinking water (control group). *n* = 5 mice for each group; *P < 0.05, between the indicated groups.

Mice were administered daily oral doses of rosuvastatin for 12 weeks to simulate the effects of continuous rosuvastatin medication on metabolism in the human body. The rosuvastatin‐treated mice gained the same amount of weight as mice in the control group during the 12 weeks of treatment (Figure 1a; *P* = 0.99, 0.858, 0.99 between the two groups at 4, 8 and 12 weeks, respectively; one‐way ANOVA). Rosuvastatin did not influence fasting blood glucose levels at 4, 8 and 12 weeks after drug administration (Figure 1b; *P* = 0.998, 0.205 and 0.707 between the two groups at 4, 8 and 12 weeks, respectively; two‐way ANOVA). Although the fasting insulin level showed no considerable changes after rosuvastatin treatment (Figure 1c; *P* = 0.219, 0.003 and 0.013 between the two groups at 4, 8 and 12 weeks, respectively; two‐way ANOVA), the IPGTT results showed that mice treated with rosuvastatin exhibited a significantly lower glucose tolerance ability than the control mice, as evidenced by the plasma glucose level (Figure 1d; *P* = 0.621, 0.03, 0,01, 0.37 and 0.917 between the two groups at time points 15, 30, 60, 90 and 120 min, respectively; two‐way ANOVA). The plasma insulin level was measured by ELISA to confirm insulin resistance (Figure 1e; *P* = 0.87, 0.898, 0.98, 0.981 and 0.999 between the two groups at time points 0, 30, 60, 90 and 120 min, respectively; two‐way ANOVA). In the IPGTT, we found that both rosuvastatin‐treated and control mice had elevated plasma insulin levels with no significant difference. Moreover, continuous rosuvastatin administration for 12 weeks appeared to exert minimal effect on most metabolic parameters in mice, as the levels of serum cholesterol (Figure 2a; *P* = 0.70), TG (Figure 2b; *P* = 0.74), HDL‐c (Figure 2c; *P* = 0.83), free fatty acids (Figure 2d; *P* = 0.21) and BHB (Figure 2e; *P* = 0.68) showed no significant changes between the control and rosuvastatin groups. However, mice in the rosuvastatin group had significantly higher serum BCAA levels than mice in the control group (Figure 2f; *P* < 0.001). Overall, in vivo continuous administration of rosuvastatin promoted insulin resistance and increased serum BCAA levels in mice.

### 3.2 Rosuvastatin disrupts the BCAA catabolism of skeletal muscle in mice

**FIGURE 3:**
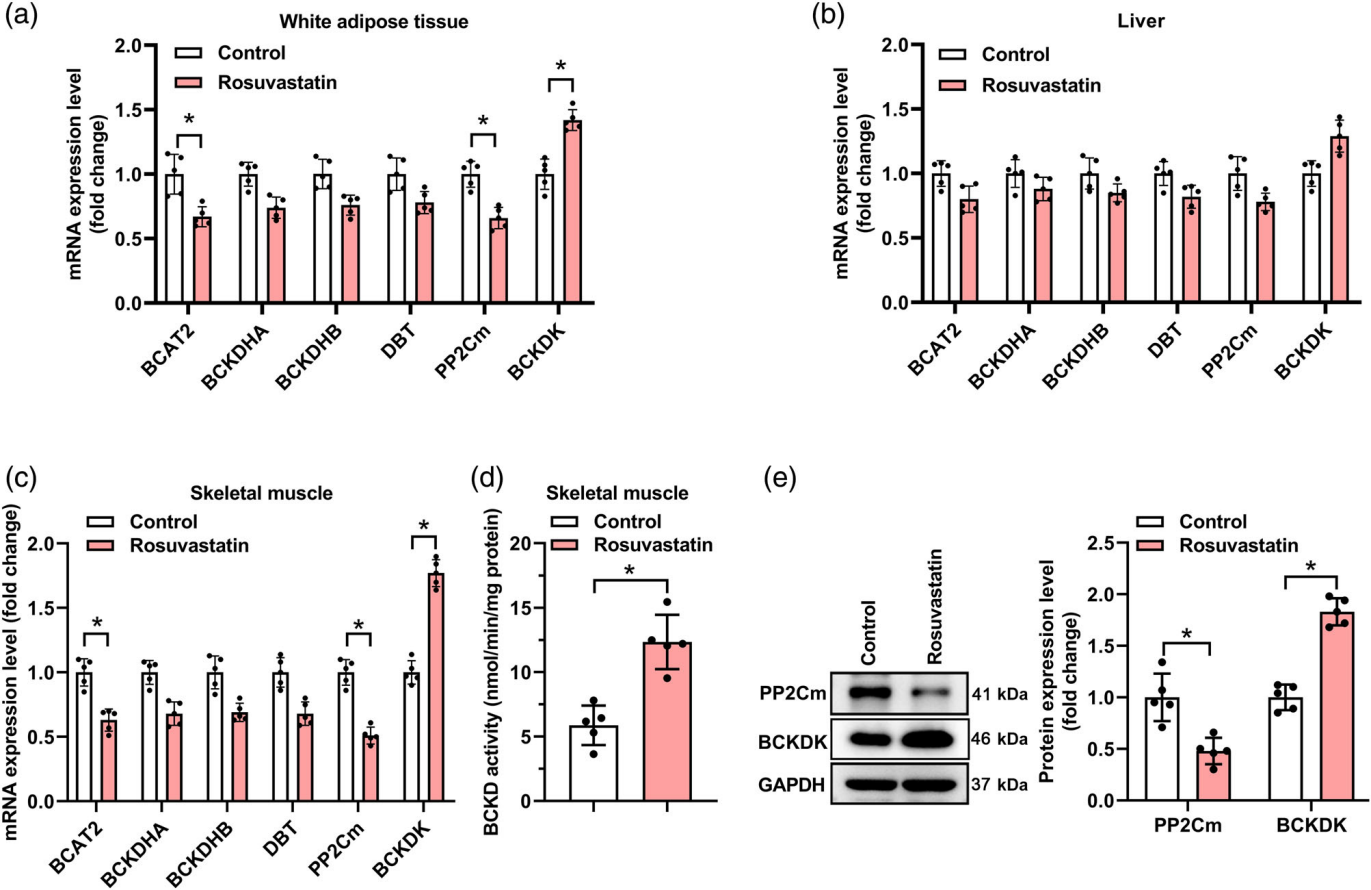
Rosuvastatin treatments in mice disrupted BCAA catabolism in the skeletal muscle. (a–e) At 12 weeks after oral dosing of rosuvastatin (rosuvastatin group) or drinking water (control group), mice were killed, and the expression levels of BCAA catabolism‐associated enzymes in multiple organs and tissues were quantified. (a–c) mRNA levels of BCAT2, BCKDHA, BCKDHB, BDT, PP2Cm and BCKDK in white adipose tissue (a), liver (b) and skeletal muscle (c) determined by qPCR assays. (d) BCKD level in the skeletal muscle from mice in the two groups. (e) Protein levels of PP2Cm and BCKDK in mouse skeletal muscle quantified by western blot assays. Representative images of blot bands are shown, and the relative protein expression levels are summarized. *n* = 5 mice for each group; *P < 0.05, between the indicated groups.

To further support the involvement of rosuvastatin in the regulation of BCAA catabolism, we investigated the expression levels of BCAA catabolism‐associated enzymes in several organs and tissues from the control and rosuvastatin‐treated mice. Compared with control mice, the white adipose tissue of rosuvastatin‐treated mice exhibited significantly reduced mRNA expression of *BCAT2* (*P* = 0.02) and *PP2Cm* (*P* = 0.03), as well as significantly higher mRNA expression of *BCKDK* (*P* = 0.04). Other genes, such as *BCKDHA* (*P* = 0.28), *BCKDHB* (*P* = 0. 20) and *DBT* (*P* = 0.11), showed no significant differences in their mRNA levels between the two groups; however, mice in the rosuvastatin group showed small declines (Figure 3a). In addition, rosuvastatin administration exerted no effect on the mRNA levels of all these genes in the liver (Figure 3b; *P* = 0.12, 0.30, 0.12, 0.11, 0.07 and 0.12 for *BCAT2*, *BCKDHA*, *BCKDHB*, *DBT*, *PP2Cm* and *BCKDK*, respectively). However, the changes in the mRNA levels of *BCAT2* (*P* = 0.02), *PP2Cm* (*P* = 0.02) and *BCKDK* (*P* = 0.01) in skeletal muscle tissues exhibited a similar pattern to that observed in the white adipose tissues of mice between the control and rosuvastatin groups (Figure 3c). Furthermore, the amount of BCKDK in the skeletal muscle tissues of rosuvastatin‐treated mice was considerably higher than that of the control mice (Figure 3d; *P* = 0.05). However, the BCKDK level in white adipose tissues remained unchanged after rosuvastatin treatment (data not shown). Moreover, consistent with the mRNA level results, the protein level of PP2Cm in skeletal muscle tissues was significantly reduced after rosuvastatin treatments (*P* = 0.02), whereas that of BCKDK was increased (*P* = 0.02), which was consistent with the results for the mRNA levels (Figure 3e). As previous studies have indicated that BCKDK is an important catabolic regulator of BCAAs (East et al., 2021; Xue et al., 2017), our findings indicated that rosuvastatin alters BCAA catabolism in the skeletal muscle tissues of mice.

### 3.3 Rosuvastatin disrupts BCAA catabolism in C2C12 myoblasts and regulates Akt and GSK3β signaling pathways

**FIGURE 4:**
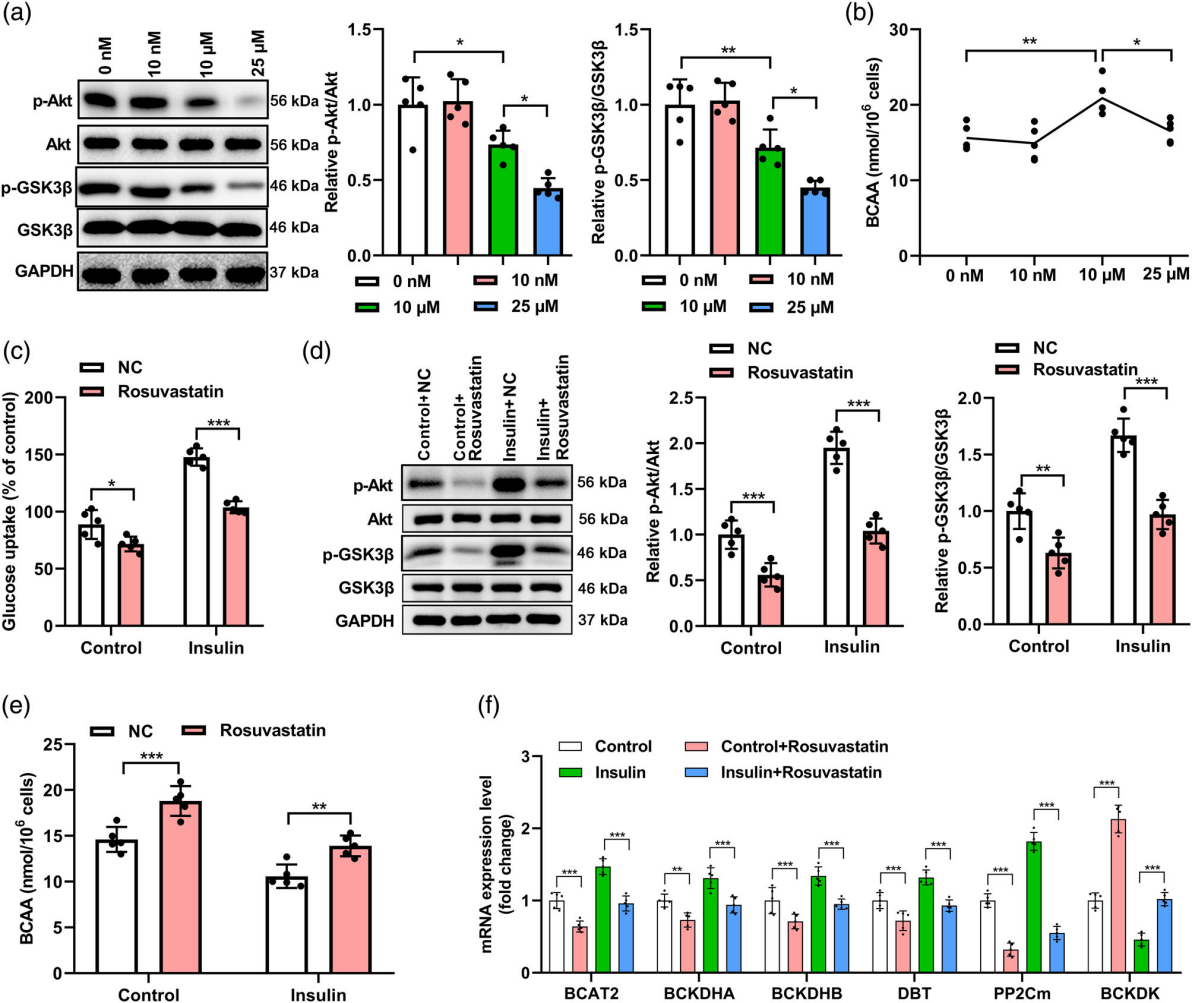
Rosuvastatin disrupted BCAA catabolism in C2C12 myoblasts. (a, b) C2C12 myoblasts were left untreated (0 nM) or treated with rosuvastatin at the concentrations of 10 nM, 10 μM or 25 μM for 48 h. The protein levels of Akt, phosphorylated Akt (p‐Akt), GSK3β and phosphorylated GSK3β (p‐GSK3β) (a) and the abundance of BCAAs (b) in C2C12 myoblasts were quantified. Representative images of blot bands are shown, and the relative ratios of p‐Akt/Akt and p‐GSK3β/GSK3β are summarized. (c–f) C2C12 myoblasts were left untreated (control) or treated with 100 nM insulin alone or 25 μM rosuvastatin alone or 100 nM insulin together with 25 μM rosuvastatin for 48 h. (c) Glucose uptake in cells of the indicated groups. Data were normalized to the value of the control group, which was set as 100%. (d) Protein levels of Akt, p‐Akt, GSK3β and p‐GSK3β in C2C12 myoblasts after the indicated treatments by western blot assay. (e) Abundance of BCAAs in C2C12 myoblasts of the indicated groups. (f) mRNA levels of BCAA catabolism‐associated enzymes in C2C12 myoblasts of the indicated groups quantified by qPCR assays. *n* = 5 for each group; *P < 0.05, **P < 0.01, ***P < 0.001 between the indicated groups.

An in vitro model was generated using C2C12 myoblasts to explore the effects of rosuvastatin on BCAA catabolism and insulin resistance‐associated signaling pathways, including Akt and glycogen synthase kinase 3 β (GSK3β) signaling (John et al., 2018; Lee & Kim, 2007; Schultze et al., 2012). First, we investigated the dose–response relationships of rosuvastatin C2C12 myoblasts. Rosuvastatin dose‐dependently inactivated Akt and GSK3β signaling (Figure 4a; for p‐Akt/Akt, *P* = 0.024, 0 nM vs. 10 μM; *P* = 0.013, 10 μM vs. 25 μM; for p‐GSK3β/GSK3β, *P* = 0.009, 0 nM vs. 10 μM; *P* = 0.015, 10 μM vs. 25 μM) and increased the BCAA levels (Figure 4b; *P* = 0.009, 0 nM vs. 10 μM; *P* = 0.015, 10 μM vs. 25 μM) in C2C12 cells when administrated at high doses of micromolar levels. However, rosuvastatin at the reported therapeutic doses in serum of patients (∼10 nM; Bjorkhem‐Bergman et al., 2011) did not elicit such evident changes. Next, the cells were treated with insulin and 25 μM rosuvastatin, either alone or in combination, and their glucose absorption was evaluated. The insulin‐treated cells exhibited considerably enhanced glucose uptake compared with control cells, whereas further rosuvastatin treatments significantly reduced the glucose uptake. Moreover, the group that received rosuvastatin treatment alone appeared to have a slightly reduced glucose absorption in C2C12 cells without statistical significance (Figure 4c; for Control, NC vs. Rosuvastin, *P* = 0.011; for Insulin, NC vs. Rosuvastin, *P* < 0.001, by two‐way ANOVA). Similarly, insulin activated the Akt and GSK3β signaling pathways, as demonstrated by elevated levels of p‐Akt and p‐GSK3β. Simultaneous treatments with rosuvastatin reversed these increases (Figure 4d; p‐Akt/Akt, for Control, NC vs. Rosuvastin, *P* < 0.001; for Insulin, NC vs. Rosuvastin, *P* < 0.001, by two‐way ANOVA; p‐GSK3β/GSK3β, for Control, NC vs. Rosuvastin, *P* = 0.0017; for Insulin, NC vs. Rosuvastin, *P* < 0.0001, by two‐way ANOVA). In addition, C2C12 myoblasts showed dramatically increased BCAA levels after rosuvastatin treatment, similar to that observed in the skeletal muscle tissues of mice. Insulin treatment reduced the BCAA levels, which were restored by adding more rosuvastatin (Figure 4e; for Control, NC vs. Rosuvastin, *P* < 0.001; for Insulin, NC vs. Rosuvastin, *P* = 0.003, by two‐way ANOVA). Moreover, similar to that observed in the skeletal muscle tissues of mice, C2C12 myoblasts showed significantly increased BCAA levels after rosuvastatin administration. Furthermore, insulin challenge in C2C12 cells dramatically elevated the mRNA levels of BCAA catabolism‐related genes, including *BCAT2* and *PP2Cm*, but significantly reduced the mRNA level of *BCKDK*, which was again reversed by additional rosuvastatin treatment. At the mRNA level, insulin challenge in C2C12 cells dramatically elevated the mRNA levels of BCAA catabolism‐related genes, including *BCAT2* (*P* < 0.001, Control vs. Insulin; *P* < 0.001, Control vs. Rosuvastatin; *P* < 0.001, Insulin vs. Rosuvastin; *P* < 0.001, Insulin vs. Insulin+Rosuvastin; *P* < 0.001, Rosuvastatin vs. Insulin+Rosuvastatin, by one‐way ANOVA), *BCKDHA* (*P* < 0.001, Control vs. Insulin; *P* = 0.002, Control vs. Rosuvastatin; *P* < 0.001, Insulin vs. Rosuvastin; *P* < 0.001, Insulin vs. Insulin+Rosuvastin; *P* = 0.002, Rosuvastatin vs. Insulin+Rosuvastatin, by one‐way ANOVA), *BCKDHB* (*P* < 0.001, Control vs. Insulin; *P* < 0.001, Control vs. Rosuvastatin; *P* < 0.001, Insulin vs. Rosuvastin; *P* < 0.001, Insulin vs. Insulin+Rosuvastin; *P*0.006, Rosuvastatin vs. Insulin+Rosuvastatin, by one‐way ANOVA), *DBT* (*P* < 0.001, Control vs. Insulin; *P* < 0.001, Control vs. Rosuvastatin; *P* < 0.001, Insulin vs. Rosuvastin; *P* < 0.001, Insulin vs. Insulin+Rosuvastin; *p* = 0.02, Rosuvastatin vs. Insulin+Rosuvastatin, by one‐way ANOVA) and *PP2Cm* (*P* < 0.001, Control vs. Insulin; *P* < 0.001, Control vs. Rosuvastatin; *P* < 0.001, Insulin vs. Rosuvastin; *P* < 0.001, Insulin vs. Insulin+Rosuvastin; *p* = 0.01, Rosuvastatin vs. Insulin+Rosuvastatin, by one‐way ANOVA), but significantly reduced the mRNA level of *BCKDK* (*P* < 0.001, Control vs. Insulin; *P* < 0.001, Control vs. Rosuvastatin; *P* < 0.001, Insulin vs. Rosuvastin; *P* < 0.001, Insulin vs. Insulin+Rosuvastin; *P* < 0.001, Rosuvastatin vs. Insulin+Rosuvastatin, by one‐way ANOVA), which was again reversed by additional rosuvastatin treatment (Figure 4f; for control vs. control+Rosuvastatin, all *P* < 0.001 except BCKDHA *P* = 0.002; for insulin vs. insulin+Rosuvastin, all *P* < 0.001, by two‐way ANOVA). Altogether, these findings suggest that rosuvastatin inhibits BCAA catabolism in C2C12 myoblasts and controls the Akt and GSK3β signaling pathways.

### 3.4 PP2Cm knockdown eradicated the effects of insulin and rosuvastatin on glucose and BCAA metabolism in C2C12 myoblasts

**FIGURE 5:**
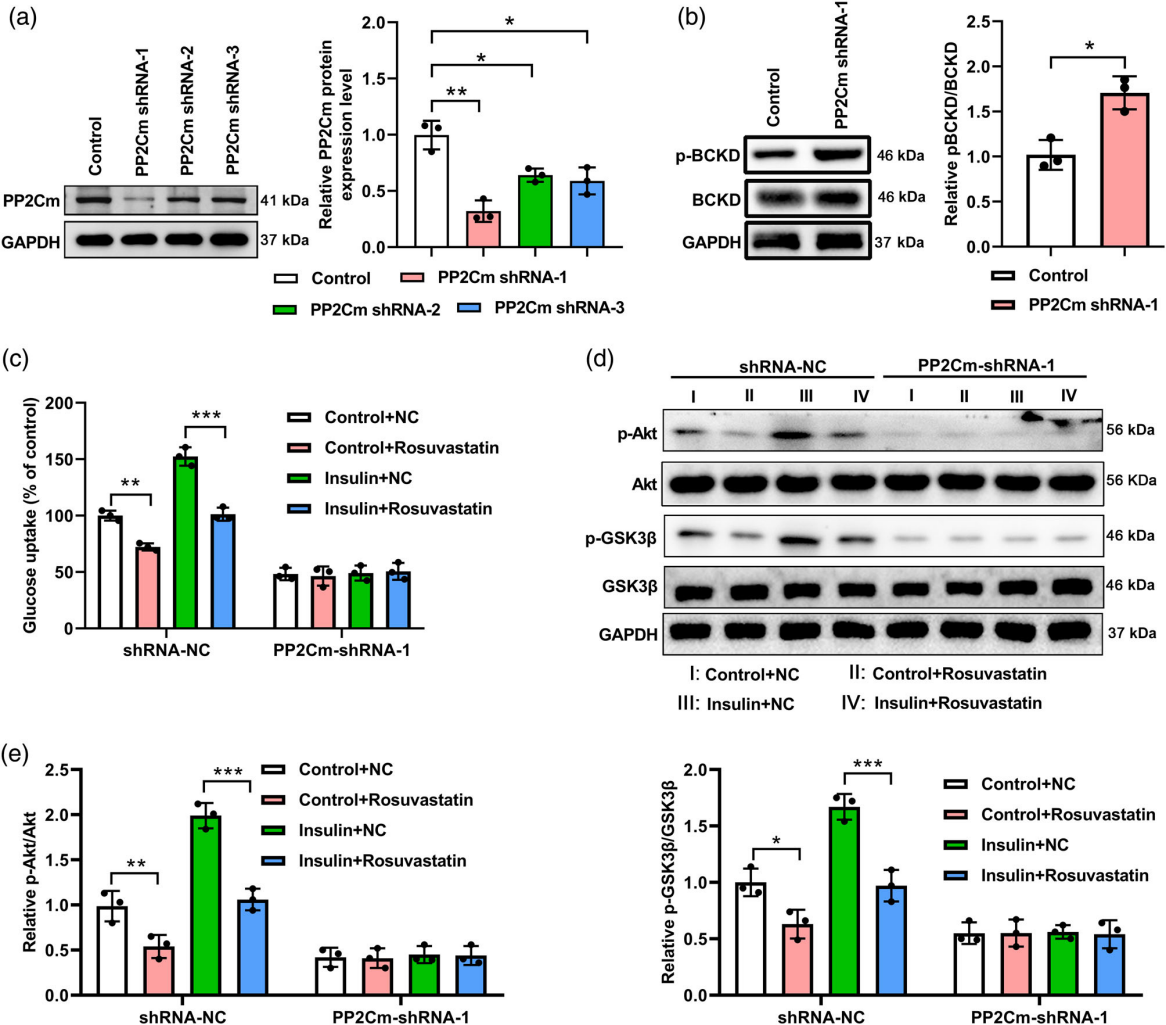
PP2Cm contributed to the effects of insulin and rosuvastatin on glucose metabolism in C2C12 myoblasts. (a) C2C12 myoblasts were infected with the shNC or PP2Cm‐specific shRNA‐expressing lentivirus, and at 48 h after virus infection, the protein levels of PP2Cm were determined by western blot assays. Representative images of blot bands and summarized relative levels of PP2Cm protein are shown. (b) Levels of BCKD and phospho‐BCKD in C2C12 myoblasts infected with the shNC or PP2Cm‐specific shRNA‐expressing lentivirus examined by western blot assays. (c–e) C2C12 myoblasts were infected with the shNC or PP2Cm shRNA‐1‐expressing lentivirus. At 24 h after virus infection, cells were further treated with PBS (control group), with insulin for 10 min, with rosuvastatin alone for 48 h, or with rosuvastatin for 48 h followed by insulin for 10 min. (c) Glucose uptake in the indicated eight groups. (d, e) Protein levels of Akt, p‐Akt, GSK3β and p‐GSK3β in C2C12 myoblasts after the indicated treatments quantified by western blot assays. Representative images of blot bands (d) and summarized relative ratios of p‐Akt/Akt and p‐GSK3β/GSK3β (e) are shown. *n* = 3 for each group; *P < 0.05, **P < 0.01, ***P < 0.001, between the indicated groups.

Both BCKDK and PP2Cm are essential regulators of BCAA catabolism. It has been reported that rosuvastatin treatment may result in increased expression of BCKDK (D'Antona et al., 2016a); however, there is limited information on the association between rosuvastatin and PP2Cm. We explored whether PP2Cm was involved in glucose control and BCAA metabolism mediated by insulin and rosuvastatin. First, we identified PP2Cm shRNA‐1 as the most effective sequence for silencing PP2Cm expression through infection using PP2Cm‐specific shRNA lentivirus and immunoblot tests (Figure 5a; *P* = 0.002, Control vs. PP2Cm shRNA‐1; *P* = 0.028, Control vs. PP2Cm shRNA‐2; *P* = 0.014, Control vs. PP2Cm shRNA‐3). Western blotting was performed to assess the amounts of BCKD and phosphor‐BCKD to confirm that PP2Cm silencing activated BCKD. Our findings indicated that the phosphorylated form of BCKD was significantly increased after the reduction of PP2Cm, although the total BCKD amount remained unaltered (Figure 5b; *P* = 0.015). Then, in parallel with the control cells, PP2Cm‐silenced C2C12 cells were either left untreated or treated with insulin and rosuvastatin, alone or in combination. The ability of insulin and rosuvastatin to regulate glucose absorption in the control cells was almost completely lost in PP2Cm‐silenced C2C12 cells (Figure 5c; for shRNA, Control+NC vs. Control+Rosuvastatin, *P* = 0.0015, Insulin+NC vs. Insulin+Rosuvastatin, *P* < 0.0001; for PP2Cm‐shRNA‐1, Control+NC vs. Control+Rosuvastatin, *P* > 0.999, Insulin+NC vs. Insulin+Rosuvastatin, *P* > 0.999, by three‐way ANOVA). Moreover, insulin treatment increased Akt and GSK3β phosphorylation in control cells but not in PP2Cm‐silenced C2C12 cells. The protein levels of Akt, p‐Akt, GSK3β and p‐GSK3β were comparable among the four groups with PP2Cm knockdown in cells (Figure 5d,e; Relative p‐Akt/Akt, for shRNA‐NC, Control+NC vs. Control+Rosuvastatin, *P* = 0.008, Insulin+NC vs. Insulin+Rosuvastatin, *P* < 0.0001; for PP2Cm‐shRNA‐1, Control+NC vs. Control+Rosuvastatin, *P* > 0.999, Insulin+NC vs. Insulin+Rosuvastatin, *P* > 0.999, by three‐way ANOVA; Relative p‐GSK3β/GSK3β, for shRNA‐NC, Control+NC vs. Control+Rosuvastatin, *P* = 0.021, Insulin+NC vs. Insulin+Rosuvastatin, *P* < 0.001; for PP2Cm‐shRNA‐1, Control+NC vs. Control+Rosuvastatin, *P* > 0.999, Insulin+NC vs. Insulin+Rosuvastatin, *P* > 0.999, by three‐way ANOVA).

## 4 DISCUSSION

Previous studies have suggested a link between rosuvastatin treatment and the development of new‐onset diabetes (Ostrowska et al., 2015; Salunkhe et al., 2016; Yoon & Lee, 2013); however, the exact processes underlying this adverse effect of rosuvastatin remain unclear. We conducted detailed in vivo and in vitro investigations using a mouse model and C2C12 myoblast cell model, respectively, to better understand the cellular and molecular mechanisms underlying the effect of rosuvastatin on glucose homeostasis. We discovered that continuous administration of rosuvastatin to mice for 12 weeks significantly reduced the intraperitoneal glucose tolerance and elevated the serum BCAA levels. Moreover, in the white adipose tissues and skeletal muscle tissues of mice treated with rosuvastatin, the accumulation of plasma BCAAs was accompanied with significantly downregulated expression of BCAT2 and PP2Cm (enzymes that enhance BCAA catabolism), as well as an increased expression of BCKDK (an enzyme that suppresses BCAA catabolism). Moreover, rosuvastatin treatment increased the BCAA levels and reduced glucose absorption in C2C12 myoblasts, which was associated with the ability of rosuvastatin to inactivate the Akt and GSK3β signaling pathways. In particular, PP2Cm expression was found to be essential in the regulation of BCAA catabolism and glucose metabolism by rosuvastatin and insulin in C2C12 myoblasts in vitro. Our findings indicate that high doses of rosuvastatin impair BCAA catabolism and causes systemic insulin resistance, which is associated with the PP2Cm‐mediated inhibition of Akt and GSK3β signaling pathways.

It should be noted that the doses of rosuvastatin used in the present studies (10 mg/kg in vivo and 25 μM in vitro), chosen based on the literature (Desjardins et al., 2008; Diaz‐Zagoya et al., 2021; Mayanagi et al., 2008; Tijeras‐Raballand et al., 2010; Zeybek et al., 2011), are much higher than those used in humans (0.1–1 mg/kg, Bjorkhem‐Bergman et al., 2011), and that the in vitro concentrations are several orders of magnitude greater than the concentrations of the drug reported in human plasma (1–15 nM, Bjorkhem‐Bergman et al., 2011). In this regard, the dose–response experiments showed that the lowest effective concentration of rosuvastatin in inhibiting Akt and GSK3β signaling in the mouse cell line C2C12 was 10 μM, which was ∼100‐fold higher than the expected levels in plasma of patients on statins. The reasons for the potentially different responsiveness to rosuvastatin in mice and humans will require further study, but it is clear that caution should be taken when extrapolating studies in rodents or non‐human cell lines to the clinical effects of the drug.

The effects of statins on insulin sensitivity appear to be contentious, with some studies claiming that statins improve insulin sensitivity (20 mg/daily tablet pravastatin treatment in patients with metabolic syndrome (Guclu et al., 2004); pravastatin at 10 mg/day in dyslipidaemic patients (Okada et al., 2005)) and others claiming that statins cause increased insulin resistance (atorvastatin at 10 mg/day and subsequent pravastatin at 20 mg/day in a patient with diabetes mellitus (Ohmura et al., 2005); atorvastatin at 10, 20, 40 and 80 mg/day in hypercholesterolaemic patients (Koh et al., 2010)); Other studies have claimed that statins exert no effect on insulin sensitivity (pravastatin 40 mg/day in healthy non‐diabetic patients (Gannagé‐Yared et al., 2005); a meta‐analysis in non‐diabetics (Baker et al., 2010)). Rosuvastatin (0.2 mg/mouse/day in the drinking water) was found to exert a dual effect on glucose homeostasis, enhancing insulin sensitivity, while decreasing insulin secretion in β‐cells (Salunkhe et al., 2016). In this study, we demonstrated that consecutive oral administration of rosuvastatin at a high dose of 10 mg/kg for 12 weeks resulted in a profoundly lower glucose tolerance in mice. Moreover, further rosuvastatin treatment at 25 μM in vitro significantly reduced glucose absorption in C2C12 myoblasts in response to insulin stimulation. A previous study showed that in patients with hyperlipidaemia with impaired fasting glucose, rosuvastatin medication (10, 20 and 40 mg/day) was associated with an increase in insulin resistance (Kostapanos et al., 2009), which is consistent with our findings. The discrepancies between our findings and those of other studies could be attributed to differences in experimental settings such as normal versus high‐fat diet feeding, various rosuvastatin doses and treatment durations, and the different molecules of statins used in animal experiments or clinical trials. According to experimental studies, the effect of statins on glucose metabolism may depend on the lipophilicity of their molecules. For instance, lipophilic simvastatin at 0.1−3 μg/ml was found to reduce glucose‐induced insulin secretion by rat pancreatic β‐cells by blocking cytosolic Ca^2+^ signaling (Yada et al., 1999). Rosuvastatin across a dose range of 1–80 mg/day in patients with dyslipidaemia may exhibit low rates of passive diffusion across pancreatic cell membranes due to its hydrophilicity and hepatoselectivity (Davidson, 2002). Consequently, the statin‐mediated decrease in β‐cell insulin production may not be the complete mechanism underlying the effect of rosuvastatin on glucose metabolism and insulin resistance. Furthermore, the metabolism and actions of rosuvastatin might differ between humans and rodents, as well as among different dose levels. Thus, our data generated from animal experiments and in vitro C2C12 myoblasts with high doses of rosuvastatin might have limited implication for direct translation of our findings to clinical patients undergoing treatments with the drug.

As a pathway fundamental to the development of diabetes mellitus, insulin signal transduction involves the phosphatidylinositol 3‐kinase–Akt (protein kinase B) pathway with the downstream involvement of GSK3 (Farrar et al., 2005; McManus et al., 2005). Insulin activates glycogen synthase by inducing its dephosphorylation at a cluster of C‐terminal residues (Ser641, Ser645, Ser649 and Ser653), which are phosphorylated by GSK3α and GSK3β. In the muscle, insulin can promote glycogen synthase dephosphorylation at these residues by triggering the inactivation of GSK3α and GSK3β through the phosphorylation of an N‐terminal Ser residue (Ser21 in GSK3α and Ser9 in GSK3β), which is processed by Akt (Ruzzin et al., 2005). We demonstrated that rosuvastatin counteracted the effect of insulin on Akt and GSK3β phosphorylation in C2C12 cells, implying the suppressive role of rosuvastatin in Akt and GSK3β signaling in myoblasts. The inhibition of Akt phosphorylation by rosuvastatin was associated with impaired S6 kinase phosphorylation, decreased protein synthesis and caspase activation, all of which indicated the induction of apoptosis (Bonifacio et al., 2015). A recent study demonstrated that rosuvastatin administration resulted in enhanced phosphorylation of Akt and GSK3β in the heart of rats with ischaemia–reperfusion‐induced myocardial injury (Velez et al., 2020). Therefore, the effects of rosuvastatin on the regulation of Akt and GSK3β signaling may be context‐dependent and further research is required to elucidate the detailed molecular pathways.

The metabolism of BCAAs is altered in various metabolic disorders, including type 2 diabetes mellitus and cardiovascular diseases. We found that rosuvastatin treatment significantly altered the levels of BCAA catabolism‐associated enzymes, such as BCAT2, PP2Cm and BCKDK, as well as BCKD, in both mouse white adipose tissues and skeletal muscle and C2C12 myoblasts, which probably played a major role in elevating plasma and cellular BCAA levels. Although there were no significant changes in the mRNA levels of these enzymes in the liver tissues of mice after rosuvastatin administration, there was a minor decrease in BCAT2, BCKDHA, BCKDHB, DBT and PP2Cm mRNA levels, as well as a slight increase in BCKDK mRNA levels. In particular, there was no significant difference in the mRNA levels of BCKDHA in skeletal muscles between the control and rosuvastatin treatment groups, although the expression of PPC2m in the skeletal muscle was found to be lower than that in other organs and tissues, such as the liver, heart, kidney and brain (Zhou et al., 2012). In C2C12 cells, we found that PP2Cm is essential for the metabolic effects of insulin and rosuvastatin. According to our findings, PP2Cm deficiency impairs BCAA catabolism, resulting in elevated plasma BCAA and BCKA concentrations (Lu et al., 2009). However, compared with wild‐type controls, PP2Cm‐deficient mice with a genetic deficiency in BCAA catabolism exhibited improved insulin sensitivity and glucose tolerance (Wang et al., 2019). This study suggests that the BCAA catabolic deficit exerts varied effects on glucose metabolism in obese and normal‐weight individuals as PP2Cm‐defective mice were leaner and preferred carbohydrate to lipids for energy production. We found that PP2Cm depletion resulted in decreased glucose absorption and insulin signaling (Figure 5), which we believe is related to altered BCAA metabolism, as PP2Cm is a key promoter of BCAA catabolism (Lian et al., 2020). Moreover, because factors such as race, gender, dietary patterns and gene variants contribute to the elevated BCAA level and exhibit obvious associations with insulin resistance (Zhao et al., 2016), a more comprehensive study involving animals of various bodyweight statuses, ages and feeding conditions would be more useful in elucidating the precise roles of rosuvastatin in regulating insulin sensitivity.

One limitation of this study is that we did not investigate rosuvastatin‐induced changes of metabolites other than BCAAs. A previous study reported that rosuvastatin administration could lower cholesterol levels (Ridker et al., 2009). A recent study reported that decreased low‐density lipoprotein cholesterol levels are associated with T2D prevalence (Klimentidis et al., 2020), indicating that the diabetogenic effect of lipid‐lowering medications such as statins is partially mediated by increased liver fat content. There is extensive literature implicating that fatty acids and other lipids are involved in the pathogenesis of metabolic diseases, which raises the possibility that the insulin resistance induced by rosuvastatin is driven by the combined effects of lipids and BCAAs (Newgard, 2012). The altered metabolism involving cholesterol, TG and HDL‐c was associated with an increasing risk of developing insulin resistance and type 2 diabetes mellitus (Pantoja‐Torres et al., 2019; Young et al., 2019). Therefore, it would be of great interest to further investigate the effect of rosuvastatin on the metabolic regulation and the underlying mechanisms.

To summarize, we found that high doses of rosuvastatin dramatically affected the expression of BCAA catabolism‐related enzymes in mouse skeletal muscle in vivo and in C2C12 myoblasts in vitro, resulting in insulin resistance and increased serum BCAA levels in mice. The disruption of BCAA catabolism and glucose metabolism caused by rosuvastatin was accompanied by the dysregulation of Akt and GSK3β signaling pathways, which was dependent on the expression of PP2Cm. Although further studies will be needed to determine whether these data obtained with high doses of rosuvastatin in mice are relevant to the therapeutic use of the drug in humans, the study highlights a potential mechanism for the diabetogenic effects of rosuvastatin, and suggests that BCAA catabolism could be a pharmacological target for preventing the adverse effects of rosuvastatin.

## AUTHOR CONTRIBUTIONS

Xue Bai, Jiquan Zhang and Xingzhen Long conceived and designed these experiments. Baolin Chen, Changcheng Sheng, Cailin Tang, Li Li, Jiaxing Zhang and Rui Zhang performed these experiments. Jiquan Zhang and Jiali Li analysed and interpreted the data. Xue Bai and Xingzhen Long wrote the manuscript. Xue Bai, Jiali Li and Fang Song revised the manuscript. All authors have read and approved the final version of this manuscript and agree to be accountable for all aspects of the work in ensuring that questions related to the accuracy or integrity of any part of the work are appropriately investigated and resolved. All persons designated as authors qualify for authorship, and all those who qualify for authorship are listed.

## CONFLICT OF INTEREST

The authors declare that they have no competing interests.

## Supporting information


Statistical Summary Document

## Data Availability

The datasets used and/or analysed during the present study are available from the corresponding author upon reasonable request.
